# Associations of Two Common Genetic Variants with Breast Cancer Risk in a Chinese Population: A Stratified Interaction Analysis

**DOI:** 10.1371/journal.pone.0115707

**Published:** 2014-12-22

**Authors:** Yuxiang Lin, Fangmeng Fu, Minyan Chen, Meng Huang, Chuan Wang

**Affiliations:** 1 Union Hospital School, Fujian Medical University, Fuzhou, China; 2 Department of General Surgery, Fujian Medical University Union Hospital, Fuzhou, China; 3 Fujian Center for Disease Control and Prevention, Fuzhou, China; University of North Carolina School of Medicine, United States of America

## Abstract

Recent genome-wide association studies (GWAS) have identified a series of new genetic susceptibility loci for breast cancer (BC). However, the correlations between these variants and breast cancer are still not clear. In order to explore the role of breast cancer susceptibility variants in a Southeast Chinese population, we genotyped two common SNPs at chromosome 6q25 (rs2046210) and in *TOX3* (rs4784227) in a case-control study with a total of 702 breast cancer cases and 794 healthy-controls. In addition, we also evaluated the multiple interactions among genetic variants, risk factors, and tumor subtypes. Associations of genotypes with breast cancer risk was evaluated using multivariate logistic regression to estimate odds ratios (OR) and their 95% confidence intervals (95% CI). The results indicated that both polymorphisms were significantly associated with the risk of breast cancer, with per allele OR = 1.35, (95%CI = 1.17–1.57) for rs2046210 and per allele OR = 1.24 (95%CI = 1.06–1.45) for rs4784227. Furthermore, in subgroup stratified analyses, we observed that the T allele of rs4784227 was significantly associated with elevated OR among postmenopausal populations (OR = 1.44, 95%CI 1.11–1.87) but not in premenopausal populations, with the heterogeneity *P* value of *P* = 0.064. These findings suggest that the genetic variants at chromosome 6q25 and in the *TOX3* gene may play important roles in breast cancer development in a Chinese population and the underlying biological mechanisms need to be further elucidated.

## Introduction

Breast cancer is one of the most common malignancies worldwide, ranking first in incidence and second in mortality among all cancers diagnosed in women. For the year 2014, it is estimated in the United States that approximately 232,670 female patients would be diagnosed with breast cancer and 40,000 would die from it [1]. And the incidence of BC is increasing rapidly in developing countries, particularly in China [2]. Breast cancer is a heterogeneous disease in which multiple environmental and genetic factors play important roles [3]. Epidemiological studies have indicated that age, obesity, a family history of BC, previous benign breast disease, menstrual and reproductive factors are associated with increased risk of BC [4]–[6]. In family-based studies, several high-penetrance inherited mutations, including BRCA1, BRCA2, TP53 and PTEN, were identified to contribute to increased susceptibility to breast cancer [7]. However, only about 25% of the familial risk and 5% of BC incidence can be explained by these high-penetrance mutations [8]–[9]. Therefore, the identification of low-penetrance genes could have a significant impact on the risk estimation of breast cancer.

In the past few years, several genome-wide association studies (GWAS) have identified a number of novel genetic susceptibility variants and loci which were independently associated with elevated risk of breast cancer [10]–[18]. Among them, two single nucleotide polymorphisms (SNPs), rs2046210 at 6q25 and rs4784227 in the *TOX3* gene were highlighted for their potential biological contribution to the development of breast cancer. SNP rs2046210 is located 180 kb upstream of estrogen receptor 1(*ESR1*) and downstream of *C6orf97*. The *ESR1* gene is of particular interest in breast carcinogenesis as it encodes estrogen receptor α (ERα). ERα regulates estrogen signal transduction and plays an important role in breast cancer [19]. The *TOX3* gene is located at chromosome 16q12.1 [10] and belongs to the large diverse family of high-mobility group (HMG) box proteins [20]. *TOX3* regulates calcium dependent transcription through the interaction with the cAMP response element binding protein [21]. In human tissues, *TOX3* is mainly expressed in the brain [22]. It is also expressed in breast, with lower levels in breast tumors than in normal tissue [23], suggesting that it may be a candidate tumor suppressor gene.

Studies by Zheng et al. [17] and Long et al. [18] first identified rs2046210 and rs4784227 as genetic susceptibility loci for breast cancer in European and Asian populations, respectively. However, several subsequent replication studies did not produce the same results. For instance, Stacy et al. [24] were unable to replicate the findings in Europeans, and similarly, Cai et al. [25] also failed to validate the associations in African Americans. Possible explanations for the conflicting results could be that study populations were different ethnic groups from different regions. Other factors may include family history or menstrual and reproductive status. Besides, tumor subtypes stratified by estrogen receptor (ER), progesterone receptor (PR) and human epidermal growth factor receptor 2 (Her-2) status may also play an important role due to different etiologic pathways. Thus, more extensive studies, especially in various populations combined with risk factors and tumor subtypes, can help us improve the understanding of genetic variants in BC etiology. Therefore, we performed a case-control study of 702 BC patients and 794 healthy controls to evaluate the associations between these two SNPs and breast cancer risk in women from southeast of China, Fujian Province. We also assessed the interactions between risk loci, traditional risk factors and specific molecular subtypes of BC defined by ER and PR status.

## Materials and Methods

### Study population

This study was a hospital-based case-control study that included 702 breast cancer patients and 794 healthy controls. All participants were genetically unrelated Chinese from Fuzhou City and its surrounding regions. Patients were consecutively recruited from the Fujian Medical University Union Hospital, Fujian, China, between June 2009 and March 2014. All BC cases were histopathologically confirmed without restriction of histological type or age. Healthy controls were frequency-matched to the BC patients by age (±5 years) and randomly selected from persons undergoing routine health examinations in the same hospital. Each participant was interviewed face-to-face by trained interviewers to gather information on demographic data, menstrual history, reproductive and breastfeeding history, previous benign breast disease history, environmental exposure history and family history of breast cancer. In addition, approximately 3 ml of venous blood was collected from each subject. Written informed consent was obtained from all participants via an institutional consent form. The study and this consent procedure were approved by the Ethics Committee of Fujian Medical University Union Hospital. The clinicopathological data of BC patients were obtained from medical records. The ER and PR status were determined by an immunohistochemical method evaluating the percentage of cancer cell nuclear staining, and the percentage of staining cells ≥10% was considered positive.

### Genotyping

Genomic DNA was extracted from the EDTA anti-coagulated whole blood using a commercially available kit according to the manufacturer's protocol (The Whole-Blood DNA Extraction Kit; Bioteke, Beijing, China). Genotyping for the two selected SNPs was performed with a custom-by-design 2×48-Plex SNPscan Kit (Cat:G0104K; Genesky Biotechnologies Inc., Shanghai, China). The kit was developed according to patented SNP genotyping technology by Genesky Biotechnologies Inc., which was based on double ligation and multiplex fluorescence PCR. Primers and probes were designed and synthesized by Invitrogen (CA, USA). Sample DNA were ligated and amplified by PCR according to the manufacturer's recommendations. The resulting data were analyzed with an ABI3730XL sequencer and GeneMapper 4.0 Software (Applied Biosystems, Foster City, CA). To ensure quality-control, genotyping was performed without knowledge of case or control status of the subjects, and approximately equal numbers of case and control samples were assayed on each 96-well plate with two blank controls. In addition, a 5% random sample of cases and controls were genotyped twice, and the concordance rate was 100%. Genotyping failed in only one breast cancer case due to DNA quality or quantity, and the average call rate for all SNPs was higher than 99%.

### Statistical analyses

Statistical analyses were performed with Statistical Package for the Social Sciences (SPSS, version 18.0) for Windows (SPSS, Chicago, IL). Differences between cases and controls in demographic characteristics, risk factors and genotype frequencies were evaluated by using χ^2^ test (for categorical variables) or Student's *t*-test (for continuous variables). The Hardy-Weinberg equilibrium (HWE) was evaluated by a goodness-of-fit χ^2^ test to compare the observed genotype frequencies with the ones in controls. Associations among genotypes, tumor subtypes and breast cancer risk were estimated by computing odds ratios (ORs) and 95% confidence intervals (CIs) from multivariate logistic regression with adjustment for age, BMI, age at menarche, age at first live birth, menopausal status and family history of breast cancer. All statistical tests were two-sided, and a *P*-value of <0.05 was considered statistically significant.

## Results

### Population characteristics

The selected characteristics compared between breast cancer and healthy-control cases are summarized in Table 1. There were no significant differences in age, BMI, menopausal status and previous benign disease between the two groups (*P*>0.05). However, compared with healthy-controls, breast cancer patients tend to have an earlier age at menarche, an earlier age at first live birth and a higher proportion of family history of breast cancer (*P*<0.05). Among 701 breast cancer cases, 479 (68.3%) cases were ER positive and 442 (63.1%) were PR positive.

**Table 1:** Distributions of selected characteristics in breast cancer cases and healthy-control cases.

Characteristics	Cases(n = 701) no.(%)	Controls(n = 794) no.(%)	*P*
Age, year(mean ± SD)	47.8±10.9	48.7±9.7	0.081
BMI, kg/m^2^(mean ± SD)	23.0±2.7	23.2±2.8	0.210
Age at menarche, y(mean ± SD)	15.2±1.6	15.5±1.7	0.001
Age at first live birth, y(mean ± SD)	24.6±3.7	25.0±3.7	0.033
Menopausal status			0.051
Premenopausal	443 (63.2)	452 (56.9)	
Postmenopausal	251 (35.8)	316 (39.8)	
Unnatural menopause^a^	7 (1)	26 (3.3)	
Previous benign breast disease			0.166
Yes	679 (96.9)	778 (98.0)	
No	22 (3.1)	16 (2.0)	
Family history of breast cancer			<0.001
Yes	51 (7.3)	10 (1.3)	
No	650 (92.7)	784 (98.7)	
ER status			
Positive	479 (68.3)		
Negative	222 (31.7)		
PR status			
Positive	442 (63.1)		
Negative	259 (36.9)		
HER-2 status			
Positive	467 (66.6)		
Negative	234 (33.4)		

a Unnatural menopause include hysterectomy operation and other status.

### Associations between SNP genotypes and breast cancer risk

The allele and genotype distribution of rs2046210 and rs4784227 in cases and controls are shown in Table 2. The observed genotype frequencies for the two SNPs were all in Hardy-Weinberg equilibrium in the control group, *P* = 0.49 for rs2046210 and *P* = 0.94 for rs4784227. In the single locus analyses, both polymorphisms achieved significant differences in the genotype distribution between cases and controls, with per allele OR = 1.35 (95%CI = 1.17–1.57) for rs2046210 and per allele OR = 1.24 (95%CI = 1.06–1.45) for rs4784227. Multivariate logistic regression analyses also revealed that, for rs2046210, the GA or AA carriers were at higher risk of BC compared with the GG homozygotes (OR = 1.63, 95%CI = 1.29–2.13 and OR = 1.68, 95%CI = 1.21–2.33, respectively). Similarly for rs4784227, in the dominant model, a significantly increased risk was observed in the CT+TT genotype, as compared to the CC genotype (OR = 1.27, 95%CI = 1.03–1.56), indicating the CT or TT carriers were associated with an altered risk of breast cancer compared with the CC homozygotes. In addition, the associations for variant genotypes in the two polymorphisms were both dose-independent, *P* trend<0.001 for rs2046210 and *P* trend = 0.009 for rs4784227.

**Table 2:** Logistic regression analyses on associations among rs2046210, rs4784227 and risk of breast cancer.

SNP	Genotype	Cases(n = 701) no.(%)	Controls(n = 794) no.(%)	*P*	Adjusted OR(95%CI)^b^	*P* trend^c^
rs2046210						
G/A^a^	GG	200 (28.5)	320 (40.3)		1.00	
	GA	382 (54.5)	361 (45.5)		1.63 (1.29–2.13)	
	AA	119 (17.0)	113 (14.2)		1.68 (1.21–2.33)	<0.001
	A allele frequency	0.442	0.370	4.1×10^−5d^	1.35 (1.17–1.57)	
	GA+AA	501 (71.5)	474 (59.7)	1.8×10^−6^ e	1.66 (1.32–2.10)	
rs4784227						
C/T^a^	CC	331 (47.2)	424 (53.4)		1.00	
	CT	302 (43.1)	313 (39.4)		1.24 (0.98–1.54)	
	TT	68 (9.7)	57 (7.2)		1.46 (0.98–2.15)	0.009
	T allele frequency	0.312	0.269	0.009^d^	1.24 (1.06–1.45)	
	CT+TT	370 (52.3)	370 (46.6)	0.032^e^	1.27 (1.03–1.56)	

a Reference allele/risk allele.

b Adjusted by age, BMI, age at menarche, age at first live birth, menopausal status and family history of breast cancer where appropriate.

c *P* trend for genotypes between cases and controls.

d Two-sided χ^2^ test for differences in frequency distribution of alleles between cases and controls.

e Two-sided χ^2^ test for differences in frequency distribution of combined genotypes(dominant model) between cases and controls.

### Associations between SNPs and breast cancer characteristics

To further evaluate the suggestive association between two polymorphisms and breast cancer risk, we performed subgroup stratified analyses according to different epidemiological characters and tumor subtypes. As shown in Table 3, scores of 0, 1 and 2 were assigned to the genotype GG, GA and AA for rs2046210 and CC, CT and TT for rs4784227 respectively (Additive model). The pooled ORs and 95% CIs were calculated in logistic regression analyses counting genotypes as ranking variables. For the rs2046210-A allele, significantly increased risks of breast cancer were consistently observed at different ages, menopausal status, age at first birth, and ER/PR subgroups. However, in the age at menarche subgroups, a significantly increased risk of breast cancer was found in later menarche individuals (OR = 1.50, 95%CI = 1.20–1.87) but not in earlier menarche individuals (OR = 1.25, 95%CI = 0.99–1.57). And no significant heterogeneity was observed between any of the stratified subgroups. For the T allele of rs4784227, positive associations with BC risk were found in postmenopausal (OR = 1.44, 95%CI = 1.11–1.87), ER positive (OR = 1.21, 95%CI = 1.02–1.42) and PR positive (OR = 1.22, 95%CI = 1.01–1.49) subjects, as compared to the premenopausal, ER negative and PR negative subgroups (OR = 1.02, 95%CI = 0.82–1.27, OR = 1.10, 0.89–1.38, and OR = 1.09, 95%CI = 0.87–1.37, respectively). In addition, a borderline *P* value (*P* = 0.064) for heterogeneity testing was observed between different menopausal subgroups, indicating rs4784227 potentially elevates a significant breast cancer risk in postmenopausal populations when compared with premenopausal populations. No significant positive associations and heterogeneity were observed in the age, age at menarche and age at first live birth subgroups.

**Table 3:** Stratified analyses on associations among rs2046210 and rs4784227 and risk of breast cancer.

Characteristics	rs2046210		OR(95%CI)^b^	*P*	*P* c	rs4784227		OR(95%CI)^b^	*P*	*P* c
	Cases(GG/GA/AA)^a^	Controls(GG/GA/AA)^a^				Cases(CC/CT/TT)^a^	Controls(CC/CT/TT)^a^			
Age										
<50	119/231/75	157/191/59	1.37 (1.11–1.70)	0.004	0.923	189/205/31	210/166/31	1.11 (0.88–1.40)	0.372	0.503
≥50	81/151/44	163/170/54	1.39 (1.10–1.77)	0.006		142/97/37	214/147/26	1.25 (0.98–1.60)	0.078	
Age at menarche										
≤15	102/214/51	157/198/58	1.25 (0.99–1.57)	0.057	0.269	169/165/33	229/153/31	1.22 (0.96–1.54)	0.097	0.639
>15	98/168/68	163/163/55	1.50 (1.20–1.87)	3.64×10^−4^		162/137/35	195/160/26	1.13 (0.88–1.43)	0.341	
Menopausal Status										
Premenopausal	120/241/82	174/214/64	1.41 (1.15–1.73)	0.001	0.647	206/206/31	232/183/37	1.02 (0.82–1.27)	0.871	0.064
Postmenopausal	77/138/36	135/135/46	1.31 (1.01–1.68)	0.038		124/90/37	178/120/18	1.44 (1.11–1.87)	0.007	
Age at first live birth										
<25	85/180/43	165/198/56	1.34 (1.06–1.69)	0.014	0.765	151/124/33	226/162/31	1.20 (0.95–1.52)	0.135	0.764
≥25	106/182/72	149/160/53	1.41 (1.13–1.74)	0.002		168/165/27	189/148/25	1.14 (0.89–1.45)	0.299	
ER status										
Positive	143/256/80		1.33 (1.11–1.58)	0.003	0.694	222/210/47		1.21 (1.01–1.42)	0.046	0.442
Negative	57/126/39		1.41 (1.12–1.77)	0.001		109/92/21		1.10 (0.89–1.38)	0.414	
PR status										
Positive	132/236/74		1.34 (1.12–1.60)	0.002	0.745	197/203/42		1.22 (1.01–1.49)	0.043	0.439
Negative	68/146/45		1.40 (1.13–1.73)	0.002		134/99/26		1.09 (0.87–1.37)	0.473	

a Counting genotypes as ranking variables.

b Derived from additive model with an adjustment by age, BMI, age at menarche, age at first live birth, menopausal status and family history breast cancer where appropriate.

c *P* for heterogeneity test.

### The combined effects of rs2046210 and rs4784227

The combined effects of the two polymorphisms are shown in Table 4. All cases and controls were categorized into five groups according to the number of risk alleles they carried (rs2046210-A and rs4784227-T). The total number of risk alleles ranged from 0 to 4 and those with 0 risk allele were regarded as the reference group. When compared to the reference group, the ORs of BC risk for individuals carrying 1, 2 and 3/4 alleles were 1.37 (95%CI = 1.01–1.88), 2.02 (95%CI = 1.45–2.82) and 1.87 (95%CI = 1.24–2.82), respectively. These suggested that individuals carrying an increased number of risk alleles can have a higher risk of breast cancer, showing a dose-dependent effect (Trend test: *P* = 1.19×10^−6^).

**Table 4:** The combined effects of rs2046210 and rs4784227.

Number of risk alleles^b^	Cases(n = 701) no.(%)	Controls(n = 794) no.(%)	OR(95%CI)^a^	*P* value
0	92	165	1	
1	266	337	1.37 (1.01–1.88)	5.36×10^−2^
2	247	207	2.02 (1.45–2.82)	3.59×10^−4^
3+4	96	85	1.87 (1.24–2.82)	2.85×10^−3^
*P* trend				1.19×10^−6^

a Adjusted by age, BMI, age at menarche, age at first live birth, menopausal status and family history breast cancer where appropriate.

b The risk allele included rs2046210-A and rs4784227-T.

## Discussion

In the present case-control study, we investigated the associations of two candidate SNPs and the risk of breast cancer in a Southeast Chinese population. We found that both rs2046210 at 6q25 and rs4784227 in the *TOX3* gene were significantly associated with increased BC risk.

Zheng et al. [17] conducted a three stage GWAS which identified rs2046210 was strongly associated with breast cancer in Asian and European populations. However, several subsequent studies did not show the same results [24], [25]. One possible reason may be the genetic differences across regions and ethnic groups, another probable explanation could be the differences in linkage disequilibrium (LD) patterns among various populations. In our studied population, rs2046210 is confirmed to be significantly associated with BC risk, which strengthens the observation that this polymorphism plays a critical role in breast cancer. In further studies, the associations between rs2046210 and BC risk appear to be similar within different subgroups while interestingly, we observed that this association was a little stronger in ER negative than in ER positive tumor subtypes. Although this difference was small and not statistically significant, it was consistent with previous reports [17], [25]. This may be due to rs2046210 being independently associated with the risk of BC for BRCA1 mutation carriers [26] while breast cancer patients with BRCA1 mutations are more often estrogen receptor negative [27].

The SNP rs2046210 lies 180 kb upstream of the *ESR1* gene. *ESR1* encodes receptor α which is activated by the hormone estrogen. Breast cancer is one of the hormone dependent malignancies and cumulative exposure to sex hormones has been suggested to be linked to the development of BC [28]. *In vitro* experiments have also proven that activating *ESR1* mutations were shown to result in continued responsiveness to anti-estrogen therapies [29].

Since the contiguous relations between rs2046210 and *ESR1*, researchers hypothesize that it was the polymorphism itself or the causal variants in LD that might regulate *ESR1* gene expression and contribute to be elevated susceptibility to breast cancer. Another SNP, rs9397435 (2.9 kb away from rs2046210) has been identified to confer BC risk to Asian, European and African populations in fine-mapping studies [24]. However, there are still no exact functional studies confirming whether rs2046210 will affect the expression of *ESR1*, thus the potential functional mechanism of this polymorphism still requires consideration and further investigation.

The SNP rs4784227 is located 18.4 kb upstream of the *TOX3* gene and in the evolutionarily-conserved portion of an intron in the *LOC643714* gene. This SNP has been implicated to be a functional genetic risk variant for breast cancer in various *in vitro* experiments. Long et al. [18] demonstrated the T risk allele could reduce luciferase activity and alter DNA-protein binding patterns. Another study showed that rs4784227 are enriched in the cistromes of *FOXA1* and *ESR1* in a cancer-cell specific manner, modulating the affinity of chromatin for *FOXA1* and resulting in allele-specific gene expression [30].

In our study, we confirmed rs4784227 as a BC susceptibility locus among a Southeast Chinese population. Further stratified analyses suggested that positive association was stronger in ER/PR positive than in ER/PR negative breast cancer which was consistent with previous data [18], [31]. In addition, we observed a meaningful result that the T allele of rs4784227 was strongly associated with breast cancer risk among postmenopausal populations (OR = 1.44, 95%CI = 1.11–1.87), but no evidence of significant associations were found in premenopausal populations (OR = 1.02, 95%CI = 0.82–1.27).

It is well established that endogenous estrogen in postmenopausal women is mainly produced by adipose tissue through the variation from androgen in the aromatase activity [32]. Circulating levels of estrogens and androgens have been demonstrated to be positively associated with the risk of breast cancer in postmenopausal women, particularly in ER positive tumor subtype [19], [33]–[34]. In some case-control studies, certain polymorphisms in fibroblast growth factor receptor 2 (*FGFR2*) and methionine synthase reductase (*MTRR*) were indicated to elevate individual susceptibility in postmenopausal breast cancer [35]–[37]. The *FGFR2* gene was identified to impact carcinogenesis through cellular signal transduction [38]–[40], while the *MTRR* gene mainly influenced folate metabolism and played important roles in DNA methylation and synthesis [37]. Furthermore, a multi-variant analysis studying genes in the estrogen metabolic pathway revealed that the association was mainly focused on polymorphisms of the androgen-to-estrogen conversion sub-pathway, and this association was confined to postmenopausal women with sporadic estrogen receptor positive tumors [41]. Considering these results, we hypothesize that the SNP rs4784227 may act as an important transcription factor in the androgen-to-estrogen conversion sub-pathway, which could result in longer estrogen exposure for postmenopausal women and increased risk of BC. And this may also partly explain why rs4784227 is more associated with ER/PR positive breast cancer. However, this is only one of the speculations about possible biological mechanisms between rs4784227 and breast cancer and needs to be confirmed by follow-up studies.

In conclusions, the present study confirmed that SNP rs2046210 and rs4784227 contributed to increased breast cancer susceptibility among a Southeast Chinese population. Moreover, our data provided additional evidence for the correlations among genetic variants, risk factors and tumor molecular subtypes. One main limitation of this study was that the sample size was still not large enough which can impact on the precision and accuracy of results, some epidemiological characteristics were also unable to be conducted in the stratified analyses. Meanwhile the functions of these two SNPs remain still unclear. Therefore, it is necessary that future larger ethnic-matched studies should be warranted and further investigations into potential biological mechanisms of these two polymorphisms are also needed.
